# The hypothalamic–pituitary–adrenal axis and sex hormones in chronic stress and obesity: pathophysiological and clinical aspects

**DOI:** 10.1111/j.1749-6632.2012.06569.x

**Published:** 2012-05-21

**Authors:** Renato Pasquali

**Affiliations:** Division of Endocrinology, Department of Clinical Medicine, S. Orsola-Malpighi Hospital, University Alma Mater Studiorum of BolognaBologna, Italy

**Keywords:** cortisol, androgens, stress, obesity

## Abstract

Obesity, particularly the abdominal phenotype, has been ascribed to an individual maladaptation to chronic environmental stress exposure mediated by a dysregulation of related neuroendocrine axes. Alterations in the control and action of the hypothalamic–pituitary–adrenal axis play a major role in this context, with the participation of the sympathetic nervous system. The ability to adapt to chronic stress may differ according to sex, with specific pathophysiological events leading to the development of stress-related chronic diseases. This seems to be influenced by the regulatory effects of sex hormones, particularly androgens. Stress may also disrupt the control of feeding, with some differences according to sex. Finally, the amount of experimental data in both animals and humans may help to shed more light on specific phenotypes of obesity, strictly related to the chronic exposure to stress. This challenge may potentially imply a different pathophysiological perspective and, possibly, a specific treatment.

## Introduction

Obesity, particularly its abdominal phenotype, is a key component of the metabolic syndrome, and represents an important risk factor for cardiovascular diseases (CVD) and type 2 diabetes (T2D).^1^,^2^ Major pathophysiological events responsible for the association between obesity and metabolic or cardiovascular dysfunctions are likely to be the development of an insulin resistance state and systemic low-grade inflammation, which are characterized by mutual feeding.^3^,^4^

There is increasing evidence that chronic environmental stressors may play an important role in increasing individual susceptibility towards the development of chronic metabolic diseases, such as abdominal obesity and the metabolic syndrome,^5^ as a consequence of an inadequate response to repeated or chronic stress stimuli and consequent whole body maladaptative behavior, according to the pioneeristic view of H Selye.^6^ In fact, stress activates the hypothalamic–pituitary–adrenal (HPA) axis, the sympathetic nervous system (SNS), and the sympathoadrenal system.^7^ Defense reactions involve the release of endogenous mediators, chiefly cortisol and catecholamines, and activate other endocrine systems, such as the renin–angiotension system and others.^8^ In conditions of continuous stress exposure and poor coping, this hormonal response may be disrupted, making the response to stress inadequate and activating compensatory mechanisms. As a consequence, the allostatic load (the body price required for adaptation) may become overwhelming and the reactive processes may be maladaptive.^9^

These concepts were reinforced by the recent finding that glucocorticoids also play a dominant role in regulating stress-induced food intake and choice by interacting, in a feedback circuit, at the central brain neuroendocrine level.^10^,^11^ Moreover, the ability to adapt to both internal and particularly to environmental (external) stressors may differ between males and females, with specific pathophysiological events leading to the development of stress-related chronic diseases.^12^ This seems to be mediated, at least in part, by the regulatory role of sex hormones on the activity of other hormonal systems, particularly the HPA axis, because a close crosstalk exists between sex hormones and glucocorticoids at both neuroendocrine and peripheral levels, with different specificities according to sex.^13^

## Neuroendocrinology of stress response: roles of HPA axis and SNS

Appropriate regulatory control of the HPA stress axis is essential to health and survival. The parvocellular cells of the paraventricular nucleus (PVN) of the hypothalamus are the major information junction for the neuroendocrine response to stressors. There is a hierarchical organization of the stress-responsive neurocircuitries on the PVN, which is able to integrate information from multiple limbic sources with internally generated and peripherally sensed information, thereby tuning the relative activity of the adrenal cortex.^14^ Corticotrophin-releasing hormone (CRH) neurons are regulated by sensory afferents that are relayed via brain-stem loci and transmit reactive stimuli that are generally excitatory and relatively direct. Conversely, limbic forebrain structures are hypothesized to convey anticipatory signals that involve processing within pathways proximal to the levels of the PVN and several hypothalamic regions. The outflow of the HPA axis is therefore a summation of integrated inputs from several forebrain regions, including the hippocampus, prefrontal cortex, amygdala, and septum. Evidence of this summation may be appreciated by the fact that many stressors produce parallel activation among numerous HPA-regulatory limbic regions,^15^,^16^ and lesions to different regions can produce similar effects on stress response.^17^ These networks are integrated in the PVN. In most organisms, the system efficiently modulates the HPA axis in accordance with needs, but there is considerable individual variation in the HPA response disposition in addition to some influence of genetic factors and early-life experience.^14^

In the hypothalamic areas, the cascade is, in turn, chiefly regulated by the CRH and arginine vasopressin (AVP),^18^ whose release activates proopiomelanocortin in anterior pituitary corticotroph cells and the release of adrenocorticotrophic hormone (ACTH) into peripheral blood, from where it targets receptors in the adrenal cortex to release glucocorticoid hormones. CRH, the pivotal signaling hypothalamic molecule in the stress response, is under the control of several complex processes, For example, CRH gene regulation involves multiple activating and repressing transcription factors, specifically the glucocorticoid receptors and cyclic AMP.^19^ Another important biological aspect is timing in the transcription of the signals, which is critical for effective glucocorticoid repression of the cyclic AMP-induced CRH gene. Recent studies performed in At-T20 cell lines have shown that a critical time window exists for effective repression of the CRH gene by glucocorticoids, whose disruption may result in a significant loss of glucocorticoid receptor-mediated repression, depending on specific situations.^20^ Interestingly, similar interactions have been described for the secretion of ACTH from the pituitary.^21^ Therefore, differences in timing of stimulatory and repression signals may be of consequence of adaptation of the organism to stress, thereby providing a molecular explanation for the variability in adaptation to stress. Additional data have shown that pulse frequency is increased under states of chronic stress in rats with genetically determined hyperresponsiveness of the HPA axis.^21^

Hyperactivation of the SNS also plays an important role in the body's response to both acute and chronic stress.^7^ The complex physiological processes by which the SNS regulates the HPA axis at various levels in the suprahypothalamic and hypothalamic and pituitary have been extensively investigated in the past and there are excellent review articles summarizing the specific role of the SNS as a mediator of the stress response and its ability to increase the activity of the HPA axis.^7^,^9^,^22^,^23^

Finally, as mentioned above and briefly discussed below, the response of the HPA axis to chronic stress is under the control of sex hormones, including androgens and estrogens.^12^,^13^,^24^ Overall, the complex interaction between different regulatory hormonal networks needs to be taken into consideration when investigating and interpreting how adaptive mechanisms to both acute and particularly chronic stress may be disrupted. On the other hand, we are facing an extraordinary challenge in the near future to better understand this complexity by using neuroimaging techniques and molecular biology, which should be integrated with a clinical setting.

## Pathophysiological aspects of stress response and obesity in humans: from Bjorntorp's hypothesis onwards

Studies performed in animal models have clearly demonstrated the dominant role of a hyperactivated HPA axis on the development of obesity and associated dysmetabolic and cardiovascular comorbidities. Excess cortisol increases lipoprotein lipase levels (a lipid-storing enzyme) in adipose tissue and particularly in the visceral fat.^25^ A series of studies performed in primates by Shively *et al.* provided an excellent model to investigate the responsibility of chronic stress in determining visceral obesity and associated metabolic and cardiovascular comorbidities.^26^–^28^ Specifically, compared to nonstressed individuals, these authors found that primates (cynomolgus monkeys) exposed for two years to chronic physical and psychological stress developed pathological behavioral changes (aggressiveness), increased body weight and visceral fat deposition, insulin resistance and hyperinsulinemia, impaired glucose tolerance or diabetes, dyslipidemia, adrenal hypertrophy, increased cortisol response to ACTH stimulation test, and coronary atherosclerosis. Clinical and epidemiological studies in humans have shown that increased HPA axis activity together with activation of the SNS may be significantly related to long-term adverse stressful events during the life span,^7^,^9^,^29^,^30^ and that the abdominal obesity phenotype may be strongly associated with stress-related or adverse life events and psychosocial conditions, low occupational and educational status, smoking habits, alcohol and/or drug abuse, fat overfeeding, psychiatric disorders, negative personality traits, and subjective abnormally perceived stress.^5^,^31^,^32^ Other studies have shown that a mild increase in cortisol levels may occur in situations such as work stress and unemployment or poor demographic conditions.^33^ A series of pathophysiological studies provided additional evidence documenting that subtle alterations of the HPA axis can be detected in abdominal obesity^34^ (see further paragraph). Overall, these data suggest a central neuroendocrine dysregulation resulting, in turn, in slightly abnormal net cortisol production, either continuous or episodic.

*Allostatic load* conceptualizes the cumulative biological burden exacted on the body through attempts to adapt to life's demands, and refers to the price the body pays for being forced to adapt to adverse environmental stressors.^9^,^22^,^35^ An individual's behavior can increase or decrease further risk for harm or disease, particularly in the long run. Behavior itself and cognition play an important role in determining what is stressful, which underlines the significant differences in how they respond to stressful situations. Key factors in this behavioral response include the interpretation of an event, differences in bodily conditions and, finally, the choices of compensation to stress. Therefore, the brain can also determine the behaviors and habits that make life more or less dangerous to the individual and may increase allostatic load in the long-term. Notably, several genetic factors have been shown to play a role in stress-related disorders and allostatic load.^35^ Derangements in the hormonal mediators are key factors in the maladaptation process to chronic stress exposure and, in turn, may be deeply involved in the pathophysiology of several chronic diseases. Using a multisystem summary measurement of the allostatic load (including measures of blood pressure, metabolic functions, and hormonal parameters, chiefly cortisol and urinary norepinephrine and epinephrine) in a large sample of elderly individuals followed for seven years, Seeman *et al.*^36^ found that higher baseline allostatic load scores were associated with significantly increased risk for seven-year mortality and with declines in cognitive and physical functioning, and were marginally associated with incident CVD events, independent of standard sociodemographic characteristics and baseline health status. Moreover, they found that the measure of allostatic load was a better predictor of mortality and decline in physical functioning than the metabolic syndrome. These findings have been extended to other inflammatory biomarkers,^37^ suggesting that these should possibly be included in the measurement of pathological allostatic load. Overall, these findings suggest that allostatic load should be carefully considered as a potential risk factor for chronic metabolic and CVD and emphasize the need for further extensive research in this area.

## Measurements of the activity of the HPA axis in obesity and the metabolic syndrome

The usual diagnostic procedures for investigating endogenous hypercortisolism in patients with Cushing's syndrome can be applied in obese dysmetabolic patients, although their sensitivity and specificity seem to be significantly reduced when dealing with subtle abnormalities of cortisol homeostasis.^38^ With these limitations, a series of studies performed in either epidemiological or clinical settings have investigated ACTH and cortisol concentrations in basal conditions—with repeated measurements to investigate daily chronobiological changes, during dynamic studies following stimulation with different neuropeptides or psychological stress tests, or by suppression with dexamethasone—to challenge the responsiveness to the inhibitory feedback system.^34^ In addition, there are studies evaluating glucocorticoid receptor density in different tissues, including adipose tissue. Finally, a growing amount of research in the last decade has focused on peripheral cortisol metabolism, particularly in the visceral fat tissues and liver, which specifically depends on the activity of enzymes controlling cortisol metabolism and reactivation from inactive compounds (cortisone), which include both 5α- and 5β-reductase and 11β-hydroxysteroid dehydrogenase type1 (11β-HSD1), respectively. These aspects have been extensively reviewed in recent years.^39^–^41^ Only omental 11β-HSD1 has been independently associated with the amount of visceral, not subcutaneous, fat in women, which supports the concept that visceral fat is the major target tissue for expression of genes related to glucocorticoid action.^42^

Basal blood levels of ACTH and cortisol are usually normal in obese subjects (in fact some studies found slightly lower morning cortisol levels), as are ACTH and cortisol daily rhythms,^43^ although some studies found either lower than normal single samples or lower 24-h integrated cortisol levels in adult obese men.^44^ One study investigated the pulsatile secretion of cortisol and ACTH during daytime in women, and showed that those with visceral obesity had higher ACTH pulse frequency and lower ACTH pulse amplitude, particularly in the morning, but similar mean ACTH basal concentrations in comparison with their gluteofemoral type obese counterpart and normal weight controls.^45^ These data may support an increased sensitivity of cortisol secretion to non-ACTH-dependent pathways, such as the peripheral noradrenergic regulatory system, particularly during the zenith phase of the daily rhythm.

The assessment of free cortisol in saliva may have a promising role in the investigation of the HPA axis,^46^ although cortisol concentrations in the saliva are approximately 30–50% lower than in the blood—its collection being noninvasive and stress free, and easy for frequent measurements, particularly for psychoneuroendocrinological and epidemiological studies.^47^ The cortisol assay may also be laboratory-independent, particularly when the LM/MS–MS technology is applied.^48^

Several studies used urinary free cortisol (UFC) excretion as an integrated measurement of daily cortisol excretion rate^43^ and reported higher than normal 24-h UFC excretion rates in women with abdominal obesity. Moreover, this measure has also been found to correlate positively with abdominal adiposity.^49^–^51^ Interestingly, UFC excretion rates during nighttime seem to be particularly useful for detecting subtle alterations of cortisol secretion rates in obese individuals.^45^ This may indirectly agree with the findings of the pulsatility study reported above, therefore suggesting that the night-time period and early morning hours are probably the best time to investigate subtle alterations of the HPA axis activity in obese individuals.

Dynamic studies in which the HPA axis was either stimulated or inhibited provide the most convincing evidence for a dysregulation of the HPA system in abdominal obesity. Specifically, there are studies demonstrating higher than normal cortisol responses after laboratory stress tests or after challenges of the HPA axis by administering CRH, or AVP alone or in combination,^34^,^52^ in women with abdominal obesity, compared to women with the peripheral phenotype, regardless of phychiatric disorders such as anxiety and depression.^53^ The strong reproducibility of the CRH test among individuals is undoubtedly a strong factor supporting the reliability of these data.^54^ Similar differences between abdominal and peripheral obese women have been reported using the ACTH stimulatory test, with either maximal^55^ or low^50^ ACTH doses. Collectively, these findings support the conclusion of there being abnormal HPA axis activity in obesity, possibly because of hypersensitized and/or hyperresponsive hypothalamic and pituitary centers, leading to slightly but inappropriately elevated net cortisol production, either continuous or episodic. Increased central activity or responsiveness of the HPA axis may in turn be dependent on activated function of the SNS. Using a slow infusion of yohimbine, an α2-adrenoreceptor antagonist (whose activation inhibits ACTH release^56^) to achieve steady-state norepinephrine plasma levels near those observed during acute stress, we found that the ACTH and cortisol responses to combined CRH and AVP challenge were reduced in normal weight controls, whereas it was significantly amplified in obese women, particularly in those with the abdominal phenotype.^57^ This suggests a specific synergy of increased noradrenergic pathways in favoring the increased responsiveness of the HPA axis to neuropeptide stimulation.

The suppressive challenge of the HPA axis using either standard or low dose dexamethasone has been investigated in the field of obesity. A standard dose (i.e., 1 mg overnight) unequivocally resulted in normal inhibition of cortisol secretion in obese individuals with different phenotypes,^58^ whereas low doses (i.e., ≤0.5 mg or less overnight) have been suggested to provide potential insights into the different responsiveness of the HPA axis and obesity (in both sexes).^59^ In this context, blunted inhibition of cortisol secretion has been reported, suggesting a reduced sensitivity to inhibition by low dose dexamethasone via downregulation of central glucocorticoid receptors.^60^ Another study randomly administered different deaxamethasone doses (0.0035, 0.0070, and 0.015 mg/kg/body weight, and a standard 1 mg dose) to both obese and normal-weight individuals. The findings showed that obesity in men did not influence hormone response to each dose, whereas in women, with increasing amounts of abdominal fat (measured by waist circumference), the suppression of cortisol tended to be significantly lower than that expected on the basis of increasing doses,^61^ further confirming the potential impairment of sensitive feedback signals in abdominally obese women.

## Human stress response: interplay between sex hormones and the HPA axis

Sex difference may exist in the response to chronic stress, particularly in those individuals susceptible to developing an abnormal allostatic load, which intrinsically implies a maladaptation (pathological) syndrome to chronic stress exposure, either internal or related to environmental factors. Intriguingly, these mechanisms may also imply derangements in the regulation of neuroendocrine and peripheral actions of sex hormones. The two systems, the HPA axis and sex hormones, may in fact mutually interact in determining the abnormal response to chronic stress, thereby favoring not only the development of the abdominal obesity phenotype, but also its association with metabolic comorbidities.^12^ Specific alterations in sex hormone secretion, transport, and metabolism are in fact present in obese males and females, and they are often associated with different patterns of body fat distribution. This topic has been extensively reviewed.^62^ In brief, obesity in males is usually associated with a parallel increase in abdominal and visceral fat, whereas in women, a clear dichotomy of fat distribution occurs, with the abdominal phenotype being characterized by an enlargement of both subcutaneous and visceral fat depots and a modest increase in fat in the gluteofemoral areas. Moreover, the increase in body weight and the development of different phenotypes of obesity is associated with sex-specific differences in the production and metabolic clearance rates of major androgens. Both sexes show a significant decrease in the synthesis of sex-hormone binding globulin (SHBG), but this may lead to an increase in free androgen blood concentrations in women with the abdominal, but not the peripheral, phenotype.^63^ Moreover, the production rates of SHBG-bound androgens (particularly testosterone) and androgens not bound to SHBG (such as dehydroepiandrosterone [DHEA] and androstenedione), have been found to be equally increased in female obesity.^64^ Therefore, women with the abdominal obesity phenotype are characterized by a *mild relative hyperandrogenic state*.^62^ Conversely, SHBG, as well as total and free testosterone blood levels, tends to progressively decrease with increasing body weight in the obese male, but 3-androstenediol glucuronide levels tend to be significantly higher, particularly in abdominally obese men, independently of circulating insulin concentrations.^62^

Sex-disease dimorphism can be observed in humans because men and women are at differential risk for a number of diseases. For example, autoimmune disorders are more common in women, whereas men are more prone to develop cardiovascular or infectious diseases.^65^ The difference between the sexes is apparent in the prevalence of common psychiatric disorders, such as depression or anxiety, which are more prevalent in women, whereas antisocial behavior or substance abuse is more common in men.^65^ Differences between the sexes in stress-related HPA axis responses have been investigated for many years, and it appears that they may not coincide with the subjective response to psychosocial stress.^66^ Notably, it should be considered that experimental design issues may be relevant in the interpretation of the data and the conclusions. Brain limbic regions are presumed to be involved in the processing of psychological stress,^67^ and there is evidence that hippocampal structures underlying higher-order cognitive processing may be sexually dimorphic,^68^ as confirmed by recent brain imaging studies.^69^,^70^

Animal studies have repeatedly shown that glucocorticoid levels are higher in females than in males,^65^ but studies in humans are still inconsistent. One of the main reasons is that the psychological approaches to investigate hormonal and metabolic dynamics associated with stress exposure have focused on acute stress tests in otherwise healthy subjects. On the other hand, data in these specific conditions support the concept that HPA axis activity, as defined in terms of cortisol response, tends to be higher in young or adult males than in their female counterparts, although contradictory results have also been reported.^65^ Conceptually, sex differences in HPA axis responses to psychological stress might differ between clinical populations with chronic diseases and healthy volunteers. A larger cortisol response to negative daily events has been found in women with major depression than in their male counterparts.^71^ By contrast, other studies have reported no sex difference or an increased HPA axis response to psychological challenges in males.^72^ Notably, most studies performed on this topic included heterogeneous populations and did not analyze sex differences.^65^ In addition, little attention has been paid to the role of genetics on the cortisol response to psychological stress, although heredity factors seemed to play a minor role in the cortisol response to psychological stress in one study.^73^ Finally, there are few data on the impact of chronic stress exposure on major chronic diseases in males and females.

**Figure 1:**
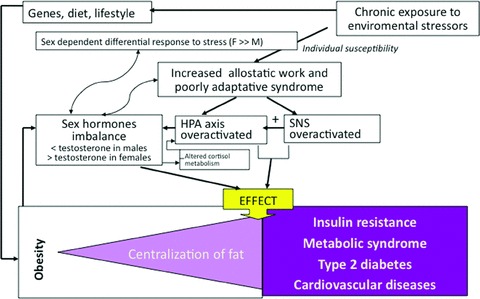
A general hypothesis on the coordinating role of the HPA axis, the SNS, and androgens in the development of central obesity and metabolic and cardiovascular comorbidities. Genes, excess diet, and unhealthy lifestyle are the main factors responsible for the development of obesity. In addition, chronic stress exposure may favor obesity by increasing allostatic work and developing a poor adaptive syndrome. Sex hormones regulate the response to stress differently in females and males. Obesity *per se* produces disparate effects on androgen production rates and metabolism according to sex. Increased *allostatic work* implies overactivation of the HPA axis and the SNS, which, in turn, further worsen androgen imbalance. The coordinating role of excess cortisol and noradrenergic tone, plus low testosterone in obese males and high testosterone in obese females, may therefore have a pathophysiological significance for the development of the abdominal phenotype of obesity and associated metabolic and cardiovascular comorbidities (from Ref. 12).

As reported above, the response to stress may be part of the biology of human sex difference, which suggests that sex hormones may be important regulators of sex-related individual responses to stress. A significant association between a list of chronic environmental stressors—such as anxiety and depressive traits (positive), alcohol abuse (positive), smoking (positive), demographic conditions (negative), occupational status (negative), educational levels (negative), personality, abuse of comfort foods (positive), and alteration of HPA axis dynamics associated with abdominal obesity—has been repeatedly described in both men and women.^30^–^32^ Moreover, an association between abdominal fatness, excess cortisol, and subjectively perceived stress (positive) has also been described in many studies performed in males or females, although such differences have not been systematically investigated. In a previous review, we emphasized the potential role of sex hormones in the relationship between alterations of the HPA axis in abdominal obesity^12^ (Fig. 1). The HPA axis is physiologically subjected to gonadal influence, as indicated by sex differences in basal and stress HPA function and by neuropathologies associated with HPA dysfunction.^14^ Although there is still no clear definition of how sex hormones mutually interact with the HPA systems,^13^ several studies performed in experimental animals have shown that a dual approach (for instance by manipulating sex hormones to evaluate effects on the HPA axis and *vice versa*) simultaneously manipulating the gonadal and adrenal axes can overcome this problem. ACTH release may be regulated by testosterone-dependent effects on AVP synthesis and by corticosterone-dependent effects on CRH synthesis in the PVN of the hypothalamus. In male rats, testosterone and corticosterone may act on stress-induced ACTH release that, ultimately, affects PVN motor neurons;^74^ and peripherally, testosterone may interfere with cortisol metabolism in the liver and the adipose tissue.^75^ In rodents, basal and stimulated ACTH and corticosterone levels tended to be greater in females than in males.^76^,^77^ Human studies have additionally found that healthy women may be more responsive to CRH, with respect to ACTH secretion.^78^–^80^ Similarly, cortisol responses to acute stress challenges have been found to be higher in abdominally obese women,^50^ and the increase in cortisol levels was found to be positively correlated with abdominal fatness in middle-aged men.^47^ We recently showed that whereas both normal-weight and obese men had significantly higher ACTH and cortisol concentrations than normal-weight and obese women, the hormone response to CRH plus AVP stimulation was conversely significantly higher in women than in men.^81^ Therefore, even in the presence of obesity, a sex difference in the activity of the HPA axis may still persist and, possibly, be amplified. However, contrasting results were reported in another study that investigated the effect of long-term gonadal suppression by leuprolide on cortisol response to CRH stimulation in healthy males and females.^82^ Additional studies performed in rats have shown that ovariectomy attenuated the response of the HPA axis, whereas estradiol replacement reversed it.^83^,^84^ Interestingly, short-term estradiol treatment led to an enhanced ACTH and cortisol stress response in young men.^85^,^86^ Another study performed in male rats found that ACTH and corticosterone responses to acute stress were increased by gonadectomy but completely reversed by testosterone replacement.^74^

Recently, one study focused on the androgen receptor mediated signaling and its interaction with corticosteroid action in an experimental model characterized by obesity, the male androgen receptor–null mutant (ARKO) mice.^86^ The authors found that the obesity state in these animals was determined by an increased corticosteroid state due to impairment of a negative feedback regulatory system. In fact, both male and female ARKO mice exhibited hypertrophic adrenal glands and glucocorticoid overproduction. This was interpreted as secondary to increased ACTH stimulation because the same study found that glucocorticoid receptor expression in a pituitary gland cell line was under the positive control of the activated ARIs (androgen receptors). This suggested that locally activated androgen receptors may support the negative feedback regulation of glucocorticoid production via upregulation of their receptor expression in the pituitary gland.^86^ These findings add significant support for the conclusion that, via their own receptors, androgens play an important role in the regulation of HPA axis activity, and that alterations of this regulatory pathway may lead to the development of obesity, at least in mice. Whether this may also occur in humans obviously needs to be defined.

**Figure 2:**
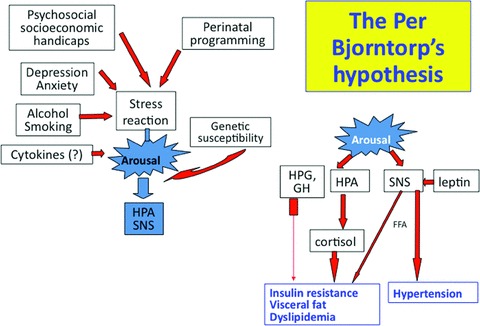
Arousal of the HPA axis and the SNS is induced by perceived stress from psychosocial and socioeconomic handicaps (related to fetal programming). Depression, anxiety, alcohol abuse, smoking (and cytokines) may cause direct activation, whereas genes may predispose for these reactions (see Refs. 29, 31).

The functional crosstalk between the HPA axis and sex steroids is, however, bidirectional, and may differ according to sex. Several years ago, Per Björntorp proposed the hypothesis that the combined alteration of the glucocorticoid pathway and androgens associated with abdominal obesity may have a role in the pathophysiology of metabolic syndrome and insulin resistance (Fig. 2).^29^ His hypothesis considered available data supporting a strict local interaction between CRH and the gonadotropin releasing hormone (GnRH),^87^ and evidence that an increased CRH secretion may inhibit GnRH secretion.^88^ On this basis, combined HOA axis hyperactivity and low testosterone levels in obese men make sense, although many other factors are involved in determining hypotestosteronemia in male obesity.^12^ Whether the same hypothesis may also apply to (abdominal) obesity in females remains unclear. As occurs in women with the polycystic ovary syndrome, there are theoretical bases to suggest that an insulin-mediated overstimulation of ovarian steroidogenesis may occur in females with abdominal obesity, as insulin acts as a true gonadotropic hormone, synergizing LH activity.^89^ However, there are no consistent *in vitro* or *in vivo* data supporting a clear responsibility of insulin—or other factors mimicking insulin action, such as the insulin growth factor-1 (IGF-1)—in women with abdominal obesity. Intriguingly, it should be considered that even in patients with Cushing's syndrome there are differences in the expression of altered gonadal function according to sex. Men with Cushing's syndrome are characterized by hypogonadotropic hypogonadism due to altered gonadotropin pulsatility, regardless of the extent of the hypercortisolism.^90^ By contrast, women with Cushing's syndrome and mild hypercortisolism may present with androgen excess of both adrenal and ovarian origin, with reduced gonadotropins and polycystic ovaries; in the case of severe hypercortisolism, the HPG axis may be inhibited, similarly to what occurs in men with Cushing's syndrome.^91^ Accordingly, in a recent study performed in obese males and females, we demonstrated a negative correlation between ACTH response to combined CRH plus AVP stimulation and testosterone, whereas cortisol response tended to be negatively correlated with the free androgen index in obese men, and positively correlated in obese women.^83^

To summarize, although there are clinical and experimental data supporting the hypothesis that the response to stress may differ to a some extent between men and women, this complex topic needs to be investigated more appropriately, possibly in selected well-defined individuals with well-defined phenotypes of obesity and chronic exposure to different stressors. In addition, the potential impact of chronic stress on eating behavior and subsequent weight changes should be investigated (see next paragraph). Finally, there is a need for prospective longitudinal studies in large cohorts of subjects of both sexes, to identify whether subtle alterations of the HPA axis (and, possibly, of the catecholaminergic system) may have a specific impact on the development of different phenotypes of obesity and associated metabolic, cardiovascular, and psychological comorbidities.

## Alterations of HPA axis activity, insulin resistance, and metabolic syndrome

Although the pathophysiology of metabolic syndrome is only partially understood, it is clear that insulin resistance has a crucial pathophysiological role in the expression of all its features, particularly abdominal obesity.^92^,^93^ Despite the lack of direct evidence in humans, several epidemiologic studies have provided evidence for a significant positive association between cortisol levels and surrogate measures of insulin resistance or metabolic alterations, other than indices of overweight or obesity.^94^,^95^ Intriguingly, some of these studies were able to detect some difference according to sex.^94^,^96^,^97^ A mild cortisol increase was identified as an early feature of essential hypertension.^98^ Interestingly, cortisol blood levels may also represent an independent risk factor for CVDs, at least in South Asian individuals, but not in Caucasians,^99^ suggesting that increased glucocorticoid action may contribute to ethnic differences in the prevalence of metabolic syndrome. Clinical studies performed in women with different obesity phenotypes have additionally shown that the cortisol response to hypothalamic neuropeptides or nightly UFC excretion rates were significantly correlated with fasting insulin or the HOMA index.^54^ Finally, the reduced allostatic load seems to be associated with lower all-cause mortality.^100^

There is also evidence for a significant association between metabolic syndrome and hyperactivity of the SNS, an important mediator, together with the HPA axis, of the stress response. Brunner *et al.*^101^ found that, compared to a nonaffected control group, middle-aged men with metabolic syndrome were characterized not only by significantly higher daily UFC, but also by increased urinary normetanephrine levels, together with more prevalent alterations of inflammatory markers and worsened indices of psychosocial distress, confirming the coordinated derangement of both major factors involved in stress adaptation and metabolic and cardiovascular abnormalities. Recently, data are emerging concerning association between the HPA axis and the occurrence of atheromatous disease.^102^ The Caerphilly Heart Study,^103^ investigating a large cohort of adult men, reported a positive association between incident CVDs and the plasma cortisol/testosterone ratio, a potential index of chronic stress exposure.^104^,^105^ A more recent prospective study in approximately 6,500 healthy men and women found that the risk of CVDs increased in relation to psychological distress, and that behavioral factors rather than pathophysiological factors explained the largest proportion of variance in defining the amount of risk.^106^ Other studies have reported that higher plasma cortisol was associated with the extent of coronary atherosclerosis, quantified by coronary angiography,^107^ or predicted mortality in patients with heart failure.^105^ Interestingly, recent epidemiological studies have also shown that polymorphisms in the glucocorticoid receptor gene may be associated with cardiovascular event rates.^108^ Given the potential importance of the pathophysiology of metabolic syndrome, the International Diabetes Federation^109^ recently recommended that a better understanding of the HPA axis function and activity in obese dysmetabolic individuals should be expanded.

## Stress, cortisol, and eating

Appetite, satiety, and reward mechanisms regulate food intake by a complex network of internal mechanisms and environmental factors. Internal factors chiefly include hormones targeting several hypothalamic nuclei,^110^,^111^ whose functions are to protect the adipose tissue stores by responding and interconnecting anorexigenic, and orexigenic signals from specific neuropeptides.^112^ In peripheral tissues, leptin and insulin are long-term signals that relay the adequacy of adipose tissue stores to the hypothalamus via the anabolic or catabolic neurons.^112^ External factors, including environmental and social conditions, and palatability of foods may in turn influence food intake.^113^ In response to acute stress, sometimes perceived as something dangerous to personal safety, a rapid physiological response is often activated,^9^ which reduces food intake by suppressing appetite.^114^ By contrast, chronic stressful situations often cause one not to avoid food but to look for energy-dense foods,^10^,^115^ which can favor rapid weight gain and lead to obesity. This depends on the magnitude, kind, and severity of events perceived as stressful, with some differences according to age and sex.^115^

Several studies have shown that women tend to increase their food consumption more frequently than males during chronic stressful conditions.^116^ The HPA axis plays an important role in the regulation of energy homeostasis and, additionally, can directly influence caloric intake, by mechanisms not fully defined, in response to stress. After chronic stress or drug-treatment with a pharmacological stressor, increased food-seeking behavior and motivation for preferred foods, together with an increase in corticosterone blood levels, have been shown in experimental animals.^117^ These changes may in turn decrease the negative impact of chronic stress on the reward pathways, depending on the action of other hormones, particularly insulin.^10^,^117^ Interestingly, compared to males, female animals may show increased stress sensitivity and display a higher hormonal response and delayed stress recovery time,^118^ suggesting that stress pathways are closely linked to brain reward centers and that some sex difference exists on the degree of reward engendered by preferred foods.

Many years ago, it was suggested for the first time that one of the contributing factors to obesity might be stress-induced eating, with a greater preference for hedonic, nutrient-dense foods, particularly those high in sugar and fat.^119^ This hypothesis has been confirmed by more recent studies, further demonstrating that individuals with high values of perceived stress are characterized by higher cortisol blood levels and a significantly higher tendency to eat more than control nonstressed individuals.^120^ This further supports the view that in vulnerable subjects, chronic stress may increase the need for increased reward functions, such as searching for energy-dense, high-calorie foods.^121^ In agreement with this, we recently demonstrated a significant positive correlation between energy intake and food choice, particularly starchy foods and daily total lipid intake, with UFC per 24 h, regardless of body weight and waist-to-hip ratio (WHR).^122^ Although much more investigation is needed in humans, these findings may provide for human obesity indirect proof of the concept of what Dallman *et al.* described in chronically stressed animals with high blood levels of corticosterone.^10^,^117^

## The SNS-neuropepdite Y network, diet, and obesity

The SNS has been implicated in the complex regulation of eating following long-term stress exposure. However, it is perplexing that not all individuals who are chronically stressed gain weight. Some people lose weight during stress, possibly because of an overactivation of the β-adrenergic lipolytic pathways.^123^ Most individuals, however, gain weight when exposed to stressful chronic events, which often seems to be disproportionate to the number of calories consumed.^124^ This may be a protective mechanism by which the body craves and stores fat because of perceived stress signals.^125^

As discussed above, an overactive HPA axis leading to elevated cortisol levels is to blame for an increased incidence of abdominal obesity, although previous studies have suggested that there are obese individuals who may have a desensitization to glucocorticoids, thereby not exhibiting increased blood cortisol.^126^ It is known that SNS activity may increase in specific phenotypes of human obesity that, in turn, may dysregulate the activity of the HPA axis and glucocorticoid action in peripheral tissues. Recent data support evidence that this may be mediated by neuropeptide Y (NPY), a sympathetic neurotransmitter, directly in adipose tissue, the effects of which may be amplified by stress, coupled with high lipid and carbohydrate feeding. In fact, a recent study performed in 129SvJ mice found that chronic stress combined with a high fat/high sugar diet led to abdominal obesity by releasing NPY directly into the adipose tissue.^127^ Moreover, the same study found that, *in vitro*, sympathetic neurons stressed with dexamethasone shifted toward expressing more NPY, which in turn stimulated endothelial cells, macrophage infiltration, and preadipocyte proliferation and lipid filling, by activation of Y2 receptors (NPY2R). Therefore, NPY is released from sympathetic nerves in animals exposed to stressors, which in turn upregulates receptors in abdominal fat in a glucocorticoid-dependent manner, thereby increasing growth and developing abdominal obesity. These findings suggest that glucocorticoids may act by priming adipose tissue for NPY effects by increasing peptide expression in sympathetic nerves. Interestingly, it was also found that the pharmacological inhibition of fat-targeted knockdown of NPY2R was antiangiogenic and antiadipogenic, with the consequence of reducing abdominal fat and associated metabolic alterations. These new findings may extend the results of Dallman's studies on the role of so-called comfort foods in the response to chronic stress, suggesting that by activating the sympathetic drive, leading in turn to peripheral NPY overexpression and action, the ingestion of high fat-high carbohydrate foods might be responsible for stress-related obesity and metabolic comorbidities. Whether this may apply to humans needs to be investigated, although some studies seem to corroborate this hypothesis. In fact, a Y2R silent mutation in a Swedish population has been associated with resistance to obesity,^128^ whereas a gain-of-function polymorphism in the NPY gene seems to predispose to individual hyperlipidemia, atherosclerosis, and severe complications of T2D.^129^ Taken together, these findings further support the metabolic impact of the SNS in the control of energy homeostasis. Among other effects, the SNS plays a major physiological role in the control of glucose metabolism, through activation of glycogen phosphorylase and inhibition of glycogen synthase, thereby increasing glycogen breakdown and glucose production, which combats the enhancement of glucose uptake by activation of the parasympathetic system.^130^ All these effects occur directly in the liver, a terminal organ of both sympathetic and parasympathetic nerves. However, these nerves are also present in various nuclei of the hypothalamus^131^–^133^ that contain many neurons expressing NPY receptors,^134^ which are in turn involved in the control of autonomic activity. In the hypothalamus, NPY stimulates the activity of the HPA axis, which in turn promotes an increase in cortisol production by stimulating gluconeogenetic pathways in different organs, particularly the liver.^135^ Recently, it was shown that intracerebroventricular administration of NPY acutely induces insulin resistance and endogenous glucose production via activation of sympathetic output to the liver.^136^ These and many other studies on the effects of increased SNS activity, mediated by both central and peripheral NPY signals, coupled with hyperactivation of the HPA axis and with the occurrence of altered feeding behaviors, may provide further pieces in the complex puzzle explaining the development of dysmetabolic obesity in subjects exposed to chronic stress.

## Does a stress-dependent obesity phenotype exist?

Based on what has been discussed above, the next step should be to translate scientific knowledge into clinics that are working to define the individual phenotype of obesity, potentially dependent on chronic stress exposure. Because this phenotype of obesity includes specific pathophysiological events, it could be theoretically possible to evaluate future strategies for intervention and treatment. Unfortunately, affected patients do not usually perceive the association between weight gain and previous stress exposure, and thus tend to ascribe their state to specific hormonal and metabolic disorders as being responsible for rapid changes in body weight and shape. On the other hand, they can easily describe their weight history during a clinical interview or understanding chat with a physician. We have described a potential phenotype of obesity related to chronic stress exposure by investigating a cohort of women who developed weight gain after a well-defined stressful event. Compared with the nonstressed group, the women were characterized by significantly higher UFC per 24-h values and a much shorter time to achieve maximum weight gain.^137^ Undoubtedly, well-defined longitudinal studies are needed to fully characterize the dynamics of weight gain and associated hormonal and metabolic alterations over time. Lessons from animal and human epidemiological and clinical studies could serve to reveal clinical, biological, and psychological aspects necessary to identify this phenotype. Other than careful personal history taking and investigating the dynamics and trajectories of weight gain, a physician should pay attention to well-defined stressful events in documenting relevant causes. Although this may be a difficult task, an appropriate investigation of eating disorders (by questionnaires, etc.), food craving patterns, or consumption of comfort foods (to improve reward pathways), particularly during the initial phases of weight gain, and any psychiatric disorders, particularly melancholic depression and the so-called stress-induced depression—a recently identified new subtype of depression^138^–^140^—should be investigated. Finally, all these data should be coupled with a detailed measurement of fat distribution and an adequate metabolic investigation. Measurement of sex hormones and simple parameters of HPA axis functioning (as discussed above) may in turn support relevant information on the pathophysiology of this specific obesity phenotype.

## Conclusions and perspectives

The existence of stress-related obesity is based on both clinical perspectives and available studies performed in experimental animals. Specific pathophysiological mechanisms may include hyperactivation of the HPA axis, which may persist if the maladaptation syndrome takes a long time to settle, and if psychiatric problems, such as depression, occur. Because of the complexity of neuroendocrine, behavioral, and metabolic adaptation to chronic stress exposure, much more research should be performed on this topic in order to plan specific therapeutic strategies.
